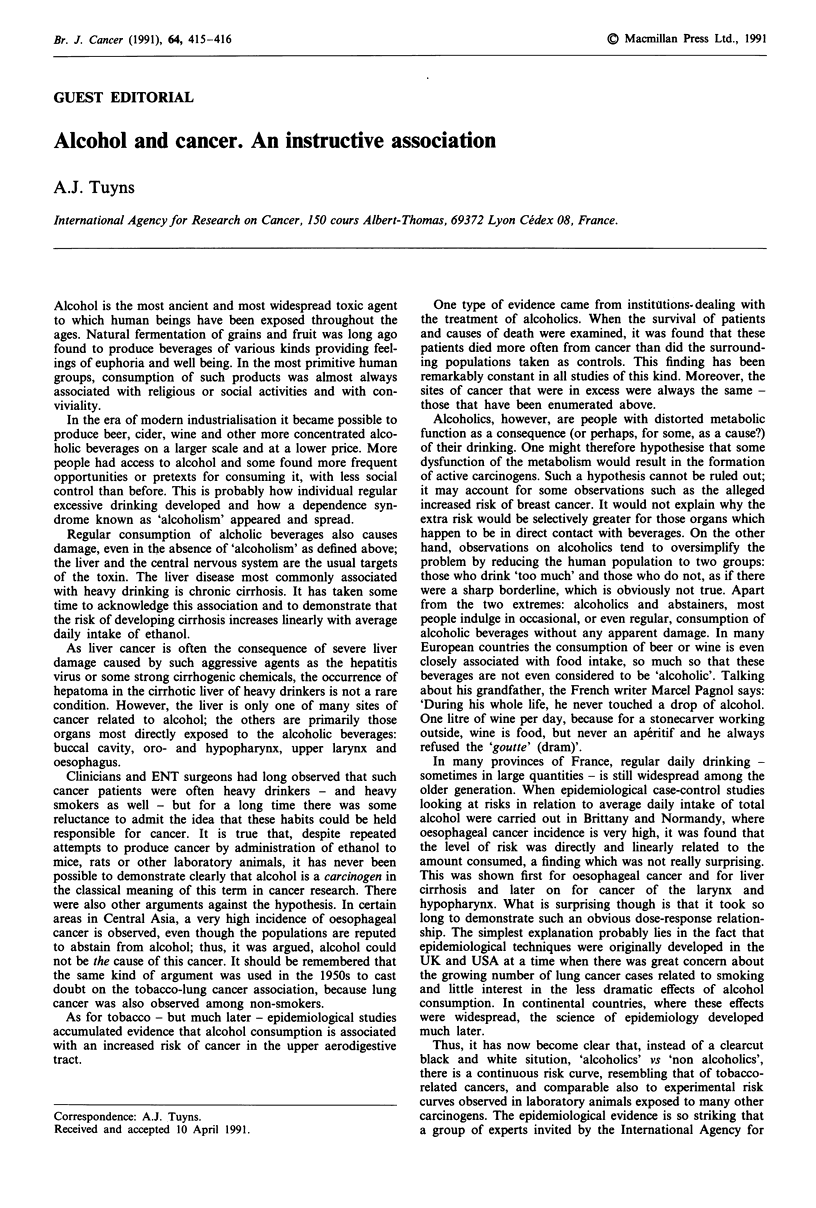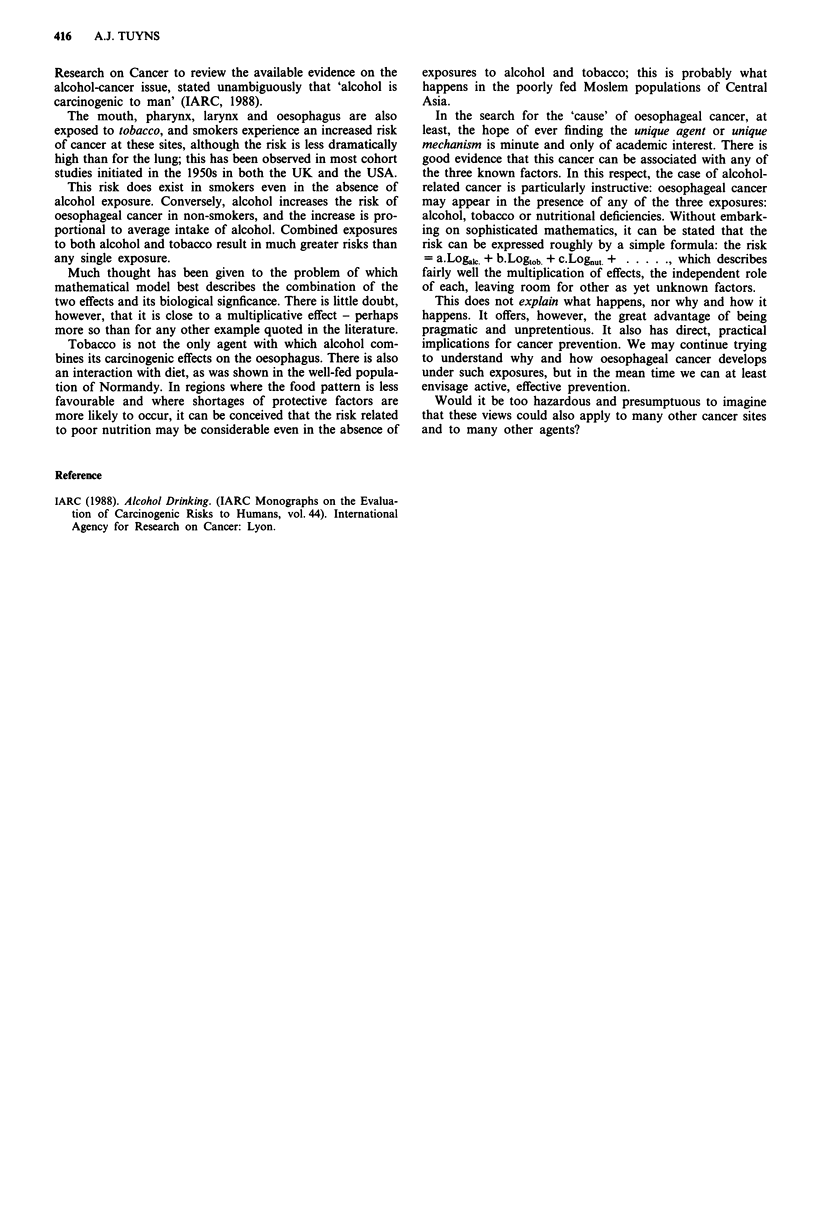# Alcohol and cancer. An instructive association.

**DOI:** 10.1038/bjc.1991.322

**Published:** 1991-09

**Authors:** A. J. Tuyns


Br. J. Cancer (1991), 64, 415–416 © Macmillan Press Ltd., 1991

GUEST EDITORIAL

### Alcohol and cancer. An instructive association

A.J. Tuyns

*International Agency for Research on Cancer, 150 cours Albert-Thomas, 69372 Lyon Cédex 08, France.*

Alcohol is the most ancient and most widespread toxic agent to which human beings have been exposed throughout the ages. Natural fermentation of grains and fruit was long ago found to produce beverages of various kinds providing feelings of euphoria and well being. In the most primitive human groups, consumption of such products was almost always associated with religious or social activities and with conviviality.

In the era of modern industrialisation it became possible to produce beer, cider, wine and other more concentrated alcoholic beverages on a larger scale and at a lower price. More people had access to alcohol and some found more frequent opportunities or pretexts for consuming it, with less social control than before. This is probably how individual regular excessive drinking developed and how a dependence syndrome known as 'alcoholism' appeared and spread.

Regular consumption of alcholic beverages also causes damage, even in the absence of 'alcoholism' as defined above; the liver and the central nervous system are the usual targets of the toxin. The liver disease most commonly associated with heavy drinking is chronic cirrhosis. It has taken some time to acknowledge this association and to demonstrate that the risk of developing cirrhosis increases linearly with average daily intake of ethanol.

As liver cancer is often the consequence of severe liver damage caused by such aggressive agents as the hepatitis virus or some strong cirrhogenic chemicals, the occurrence of hepatoma in the cirrhotic liver of heavy drinkers is not a rare condition. However, the liver is only one of many sites of cancer related to alcohol; the others are primarily those organs most directly exposed to the alcoholic beverages: buccal cavity, oro- and hypopharynx, upper larynx and oesophagus.

Clinicians and ENT surgeons had long observed that such cancer patients were often heavy drinkers – and heavy smokers as well – but for a long time there was some reluctance to admit the idea that these habits could be held responsible for cancer. It is true that, despite repeated attempts to produce cancer by administration of ethanol to mice, rats or other laboratory animals, it has never been possible to demonstrate clearly that alcohol is a *carcinogen* in the classical meaning of this term in cancer research. There were also other arguments against the hypothesis. In certain areas in Central Asia, a very high incidence of oesophageal cancer is observed, even though the populations are reputed to abstain from alcohol; thus, it was argued, alcohol could not be *the* cause of this cancer. It should be remembered that the same kind of argument was used in the 1950s to cast doubt on the tobacco-lung cancer association, because lung cancer was also observed among non-smokers.

As for tobacco – but much later – epidemiological studies accumulated evidence that alcohol consumption is associated with an increased risk of cancer in the upper aerodigestive tract.

Correspondence: A.J. Tuyns.
Received and accepted 10 April 1991.

One type of evidence came from institutions dealing with the treatment of alcoholics. When the survival of patients and causes of death were examined, it was found that these patients died more often from cancer than did the surrounding populations taken as controls. This finding has been remarkably constant in all studies of this kind. Moreover, the sites of cancer that were in excess were always the same – those that have been enumerated above.

Alcoholics, however, are people with distorted metabolic function as a consequence (or perhaps, for some, as a cause?) of their drinking. One might therefore hypothesise that some dysfunction of the metabolism would result in the formation of active carcinogens. Such a hypothesis cannot be ruled out; it may account for some observations such as the alleged increased risk of breast cancer. It would not explain why the extra risk would be selectively greater for those organs which happen to be in direct contact with beverages. On the other hand, observations on alcoholics tend to oversimplify the problem by reducing the human population to two groups: those who drink 'too much' and those who do not, as if there were a sharp borderline, which is obviously not true. Apart from the two extremes: alcoholics and abstainers, most people indulge in occasional, or even regular, consumption of alcoholic beverages without any apparent damage. In many European countries the consumption of beer or wine is even closely associated with food intake, so much so that these beverages are not even considered to be 'alcoholic'. Talking about his grandfather, the French writer Marcel Pagnol says: 'During his whole life, he never touched a drop of alcohol. One litre of wine per day, because for a stonecarver working outside, wine is food, but never an apéritif and he always refused the '*goutte*' (dram)'.

In many provinces of France, regular daily drinking – sometimes in large quantities – is still widespread among the older generation. When epidemiological case-control studies looking at risks in relation to average daily intake of total alcohol were carried out in Brittany and Normandy, where oesophageal cancer incidence is very high, it was found that the level of risk was directly and linearly related to the amount consumed, a finding which was not really surprising. This was shown first for oesophageal cancer and for liver cirrhosis and later on for cancer of the larynx and hypopharynx. What is surprising though is that it took so long to demonstrate such an obvious dose-response relationship. The simplest explanation probably lies in the fact that epidemiological techniques were originally developed in the UK and USA at a time when there was great concern about the growing number of lung cancer cases related to smoking and little interest in the less dramatic effects of alcohol consumption. In continental countries, where these effects were widespread, the science of epidemiology developed much later.

Thus, it has now become clear that, instead of a clearcut black and white sitution, 'alcoholics' *vs* 'non alcoholics', there is a continuous risk curve, resembling that of tobacco-related cancers, and comparable also to experimental risk curves observed in laboratory animals exposed to many other carcinogens. The epidemiological evidence is so striking that a group of experts invited by the International Agency for

Research on Cancer to review the available evidence on the alcohol-cancer issue, stated unambiguously that 'alcohol is carcinogenic to man' (IARC, 1988).

The mouth, pharynx, larynx and oesophagus are also exposed to *tobacco*, and smokers experience an increased risk of cancer at these sites, although the risk is less dramatically high than for the lung; this has been observed in most cohort studies initiated in the 1950s in both the UK and the USA.

This risk does exist in smokers even in the absence of alcohol exposure. Conversely, alcohol increases the risk of oesophageal cancer in non-smokers, and the increase is proportional to average intake of alcohol. Combined exposures to both alcohol and tobacco result in much greater risks than any single exposure.

Much thought has been given to the problem of which mathematical model best describes the combination of the two effects and its biological signficance. There is little doubt, however, that it is close to a multiplicative effect – perhaps more so than for any other example quoted in the literature.

Tobacco is not the only agent with which alcohol combines its carcinogenic effects on the oesophagus. There is also an interaction with diet, as was shown in the well-fed population of Normandy. In regions where the food pattern is less favourable and where shortages of protective factors are more likely to occur, it can be conceived that the risk related to poor nutrition may be considerable even in the absence of exposures to alcohol and tobacco; this is probably what happens in the poorly fed Moslem populations of Central Asia.

In the search for the 'cause' of oesophageal cancer, at least, the hope of ever finding the *unique agent* or *unique mechanism* is minute and only of academic interest. There is good evidence that this cancer can be associated with any of the three known factors. In this respect, the case of alcohol-related cancer is particularly instructive: oesophageal cancer may appear in the presence of any of the three exposures: alcohol, tobacco or nutritional deficiencies. Without embarking on sophisticated mathematics, it can be stated that the risk can be expressed roughly by a simple formula: the risk $= a.\text{Log}_{\text{alc.}} + b.\text{Log}_{\text{tob.}} + c.\text{Log}_{\text{nut.}} + \ldots\ldots$, which describes fairly well the multiplication of effects, the independent role of each, leaving room for other as yet unknown factors.

This does not *explain* what happens, nor why and how it happens. It offers, however, the great advantage of being pragmatic and unpretentious. It also has direct, practical implications for cancer prevention. We may continue trying to understand why and how oesophageal cancer develops under such exposures, but in the mean time we can at least envisage active, effective prevention.

Would it be too hazardous and presumptuous to imagine that these views could also apply to many other cancer sites and to many other agents?

### Reference

IARC (1988). *Alcohol Drinking*. (IARC Monographs on the Evaluation of Carcinogenic Risks to Humans, vol. 44). International Agency for Research on Cancer: Lyon.